# Anti-inflammatory effects of *Cordyceps* mycelium (*Paecilomyces hepiali*, CBG-CS-2) in Raw264.7 murine macrophages

**DOI:** 10.1007/s13596-014-0173-3

**Published:** 2014-12-05

**Authors:** Seong-Yeol Park, Su-Jin Jung, Ki-Chan Ha, Hong-Sig Sin, Seung-Hwan Jang, Han-Jung Chae, Soo-Wan Chae

**Affiliations:** 1 Department of Dental Pharmacology and Wonkwang Dental Research Institute, School of Dentistry, Wonkwang University, Iksan, 570-749 Republic of Korea; 2 Clinical Trial Center for Functional Foods (CTCF2), Chonbuk National University Hospital, 20, Geonjiro, Deokjin-gu, Jeonju-si, Jeollabuk-do 561-712 Republic of Korea; 3 Healthcare Claims & Management Inc., 758, Baekjedaero, Deokjin-gu, Jeonju-si, Jeollabuk-do 561-832 Republic of Korea; 4 CHEBIGEN Inc., 111-18, Wonjangdong-gil, Deokjin-gu, Jeonju-si, Jeollabuk-do 561-360 Republic of Korea; 5 Department of Pharmacology, Chonbuk National University Medical School, 567 Baekje-daero, deokjin-gu, Jeonju-si, Jeollabuk-do 561-756 Republic of Korea

**Keywords:** *Cordyceps*, *Paecilomyces hepiali*, Anti-inflammation, CBG-CS-2

## Abstract

*Cordyceps* (CS) is a traditional Chinese herb with various biological effects that include immune modulation. CBG-CS-2 is a strain, *Paecilomyces hepiali*, of the *Cordyceps* spp. The anti-inflammatory effects of CBG-CS-2 were investigated. The water-soluble fraction of CBG-CS-2 has high anti-inflammatory activity in LPS-induced Raw264.7 macrophages. We tested the role of CBG-CS-2 on the anti-inflammation cascade in LPS-stimulated Raw264.7 cells. CBG-CS-2 significantly decreased NO production, iNOS expression, and pro-inflammatory cytokine secretion in a dose-dependent manner. To investigate the mechanism by which CBG-CS-2 inhibits NO, iNOS, and pro-inflammatory cytokines, we examined the activities of NF-κB and AP-1 in LPS-activated macrophages. The results demonstrate that CBG-CS-2 suppresses the production and expression of NO, iNOS, and pro-inflammatory cytokines in LPS-activated macrophages via inhibition of NF-κB and AP-1, which may play an important role in inflammation. These findings suggest that CBG-CS-2 has modulatory effects on the inflammatory system in macrophages, and that it can serve as a useful anti-inflammatory dietary supplement or drug.

## Introduction

*Cordyceps* mycelium has long been recognized as an important medicinal mushroom in China. Its pharmaceutical properties were recorded in the book “Ben-Cao-Bei-Yao,” edited by Wang Ang in 1694. *Cordyceps* mycelium has beneficial effects on the human body, which include immune, anti-tumor, anti-metastatic, antioxidant, anti-inflammatory, insecticidal, antimicrobial, hypolipidemic, hypoglycemic, anti-aging, neuroprotective, and renoprotective effects (Paterson 2008; Zhou, Gong et al. 2009; Shin, Kwon et al. 2010). *Cordyceps* mycelium-derived natural products are comprised of complex components, including cordycepin derivatives, cordycepic acid, ergosterol, polysaccharides, and nucleosides (Li, Yang et al. 2006; Yue, Ye et al. 2013). Adenosine, cordycepin, cordycepic acid, and polysaccharides have been thought to be the main active ingredients, although this is still debated (Yue, Ye et al. 2013).

*Cordyceps* mycelium has been reported to function as an aphrodisiac (Bhattarai 1989), an analgesic (Koyama, Imaizumi et al. 1997), an immune modulator (Zhou, Gong et al. 2009), a free radical scavenger (Wang, Won et al. 2005), and an anti-cancer agent (Sun, Chia et al. 2005; Jin, Kim et al. 2008; Yoshikawa, Kunitomo et al. 2009). Because natural *Cordyceps* mycelium is rare and expensive, many scientists have examined its life cycle with the aim of developing techniques for the isolation and culture of fermentable strains.

*Paecilomyces hepiali* (PH) is a derivative of *Cordyceps sinensis* (CS), a fungus that has been shown to have anti-cancer and pro-apoptotic effects. This strain was one of the best known CS derivatives (Buenz, Bauer et al. 2005). Some studies have shown that PH can inhibit tumor proliferation, invasion, metastasis, and neovascularization; induce apoptosis; reverse drug resistance; and enhance immunity (Ng and Wang 2005; Wang, Won et al. 2005). Despite these reports on the inhibitory potential of PH on immune modulation, there have been no conclusive reports thus far on the mechanisms responsible for PH-mediated anti-inflammatory effects in macrophages.

Moreover, most of the aforementioned studies used only active ingredient extracts of mycelia. When the cultured mycelium was dissolved in water, most of the mycelium was precipitated. Only a small portion of the mycelium dissolved into the water, which is referred to as the extracted active ingredient of mycelium. Thus, the active ingredient portion was in a very highly concentrated form, relative to the total mycelium. However, for general applications of these mycelia, the water-soluble form was employed, not the highly concentrated form, as was the case for the experimental conditions.

Thus, in the present report, we examined the anti-inflammatory effects of CS mycelium (*Paecilomyces hepiali*, CBG-CS-2) using water-soluble fractions on murine macrophage Raw264.7 cells.

## Materials and methods

### Preparation of water-soluble fraction of CBG-CS-2 from *Cordyceps* mycelium (*Paecilomyces hepiali*)

Cultures of fruiting bodies of *Paecilomyces hepiali* were identified and supplied by Chebigen Inc. The dried powder of mycelium was dissolved in distilled water for 2 h at room temperature. After 2 h, the solution was centrifuged at 10,000 ×*g* for 1 min and followed by discarding of insoluble pellets. The water-soluble supernatants were filtered and named ‘the water-soluble fraction of CBG-CS-2’. The concentration of ‘the water-soluble fraction of CBG-CS-2’ used in this study is represented as the concentration obtained during the preparation of mycelium solution initially, indicated by the mark ‘S’, e.g., 500S μg/ml. For example, as we dissolved 500 μg of mycelium powder in 1 ml of distilled water and get ‘the water-soluble fraction of CBG-CS-2’ after centrifugation, the concentration of ‘the water-soluble fraction of CBG-CS-2’ was 500S μg/ml.

### Cell line and culture conditions

Mouse macrophage Raw264.7 cells were purchased from the Korean Cell Line Bank (KCLB, Korea). Raw264.7 cells were cultured in DMEM supplemented with 10 % FBS (Gibco) and antibiotics (penicillin/streptomycin) at 37 °C in a humidified culture chamber containing 5 % CO_2_.

### Crystal violet viability assay

A crystal violet assay was applied to measure cytotoxicity. Crystal violet stains the nuclei of cells. This assay identifies live cells that adhere to the culture vessel. 2 × 10^5^ cells per well were seeded with various concentrations of CBG-CS2 extract in 48-well culture plates. After overnight culture, culture medium was removed and 0.5 % crystal violet in 2 % formaldehyde was added to each well for 10 min. Excess stain and dead cells were removed by washing with tap water and the stain was dissolved using 1 % sodium dodecyl sulfate (SDS) before determining its optical density at 595 nm.

### Measurement of NO

The amount of NO produced by mouse macrophages was measured with the Griess reagent system (Promega) in cell culture supernatant. NO detection was performed according to the manufacturer’s instructions. Briefly, 50 μl of sulfanilamide solution was added to an equal volume of the cultured supernatant with various concentrations of CBS-CS2 extract and LPS. After incubation for 10 min, 50 μl of NED solution was added and incubated an additional 10 min. The value of NO production was detected as optical density at 550 nm.

### Cytokine assays

The amount of TNF-α in the culture supernatant of Raw264.7 cells was measured using a Quantikine ELISA kit (R&D Systems). The cells were treated with various concentrations of CBG-CS2 extract in the absence or presence of LPS (1 μg/ml) at 37 °C in humidified air with 5 % CO_2_. Subsequently, the supernatant of culture was assayed according to the manufacturer’s instructions.

### Western blotting

Equal amounts of each cell lysate were electrophoresed on sodium dodecyl sulfate-polyacrylamide gels (SDS-PAGE), after which resolved proteins were transferred to polyvinylidenedifluoride (PVDF) membranes. The membranes were incubated with iNOS primary antibody (Santa Cruz) diluted in TBST (20 mM Tris, 134 mM NaCl, 0.02 % Tween 20). The primary antibodies were then probed with horseradish peroxidase-conjugated secondary antibodies and visualized by exposure to an enhanced chemiluminescence reagent.

### RT–PCR for iNOS, TNF-α, and IL-6

For RT–PCR analysis, RNA was extracted using TRIzol reagent (Invitrogen Life Technologies), and complementary DNA was prepared with a SuperScript III First-Strand synthesis system (Invitrogen Life Technologies) according to the manufacturer’s instructions. The iNOS primers were: forward (5′-GCA GAA TGT GAC CAT CAT GG-3′) and reverse (5′-ACA ACC TTG GTG TTG AAG GC-3′). The TNF-α primers were: forward (5′-TAC TGA ACT TCG GGG TGA TTG GTC C-3′) and reverse (5′-CAG CCT TGT CCC TTG AAG AGA ACC-3′). The IL-6 primers were: forward (5′-CCG GAG AGG AGA CTT CAC AG-3′) and reverse (5′-GGA AAT TGG GGT AGG AAG GA-3′). Mouse iNOS, TNF-α, and IL-6 mRNA expression were quantified using i-star Taq (iNtRON Biotechnology) and their relative expression was determined by normalizing the expression of each target to mouse glyceraldehyde 3-phosphate dehydrogenase (GAPDH; forward 5′-ACC ACA GTC CAT GCC ATC AC-3′, reverse 5′-CAC CAC CCT GTT GCT GTA GCC-3′). Amplification was conducted in a total volume of 20 μl for 30 cycles of 10 s at 95 °C, 10 s at 60 °C, and 30 s at 72 °C. Samples were run in triplicate.

### Transfection and luciferase reporter assay

The response regions of the NF-κb (5′-TGGGAATTT CCGGGGACTT TCCGGGAATT TCCGGGGACTTTCCGGGAATTTCC-3′) and AP-1 (5′-TGACACA-3′) genes were subcloned into the pGL3 vector. Raw264.7 cells were plated in 10-cm culture dish to achieve 80-90 % confluence at the time of transfection. Cells were transfected using Lipofectamine 2000 transfection reagent (Invitrogen) with 30 μg of pGL3-NF-κb and pGL3-AP-1 plasmid for 24 h, then seeded to 24-well culture plate. To measure the NF-κb and AP-1 transcriptional activity reporters, cells were treated CBG-CS2 extract in the absence or presence of LPS, then firefly and Renilla luciferase activities were measured using a dual luciferase assay kit (Promega).

### Statistics

The results of the cytokine ELISA are expressed as the mean ± standard error (SEM). An F-test was used to examine variance, and the significance of differences between the LPS-treated group with and without CBG-CS-2 were determined by Student’s *t*-test according to the results of the F-test. *P* values less than 0.05 were considered to indicate significance.

## Results

### CBG-CS-2 reduced LPS-induced NO accumulation in macrophages

**Fig. 1:**
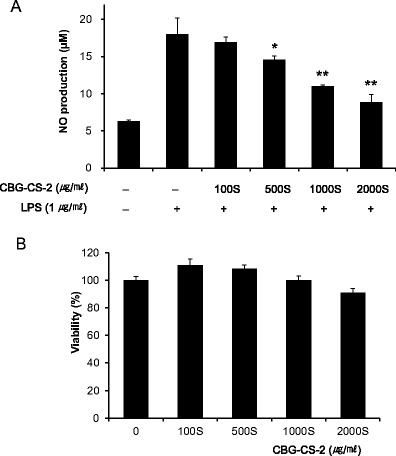
Effects of CBG-CS-2 on LPS-induced NO production in mouse macrophage Raw264.7 cells. **a** Cells were pretreated with 100S–2000S μg/ml of CBG-CS-2 before administration of LPS (1 μg/ml). Supernatants were analyzed following 20 h in culture to determine their concentrations of nitric oxide. **b** Cell cytotoxicity of CBG-CS-2 against mouse macrophage Raw264.7 cells. Cell viability was estimated via crystal violet assay. The results are expressed as mean with SEM from three independent experiments. *P < 0.05 and **P < 0.01, Student’s *t*-test

Raw264.7 macrophages were stimulated with LPS (1 μg/ml) for 20 h to induce iNOS. Co-treatment of cells with CBG-CS-2 significantly reduced NO accumulation at doses ≥500S μg/ml in Raw264.7 macrophages (Fig. 1a). CBG-CS-2, at concentrations up to 2000S μg/ml, does not decrease viability of macrophages (Fig. 1b). Thus, the inhibitory effect of CBG-CS-2 on NO synthesis was not due to any cytotoxicity of CBG-CS-2.

### CBG-CS-2 has inhibitory effects on LPS-induced mRNA and protein expression of iNOS

**Fig. 2:**
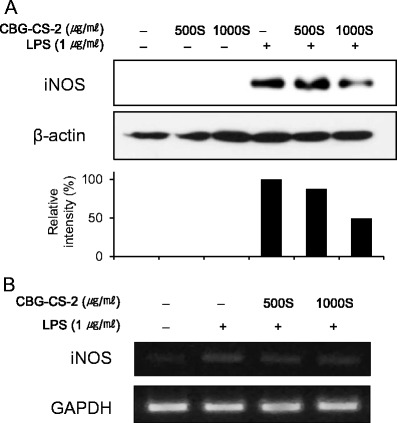
Effects of CBG-CS-2 on the expression of iNOS protein and mRNA in macrophage Raw264.7 cells. Cells were treated with two concentrations (500S and 1000S μg/ml) of CBG-CS-2, with or without LPS (1 μg/ml) for 20 h. **a** Total cellular proteins (50 μg) were separated and blots were probed with specific antibodies. **b** iNOS mRNA in Raw264.7 cells was assessed by RT-PCR. The experiments were repeated three times, and similar results were obtained

RT-PCR and western blot analyses were performed to determine whether CBG-CS-2 has a direct effect on the pro-inflammatory mediator NO related to modulation of the expression of iNOS, As seen in Fig. 2, iNOS protein expression was markedly induced in macrophage cells after treatment with LPS. This induction was decreased by CBG-CS-2 treatment in a dose-dependent manner. Furthermore, RT-PCR analysis revealed that the expression of the iNOS gene was correlated with its protein levels (Fig. 2b).

### LPS-induced TNF-α production was inhibited by CBG-CS-2 in macrophages

**Fig. 3:**
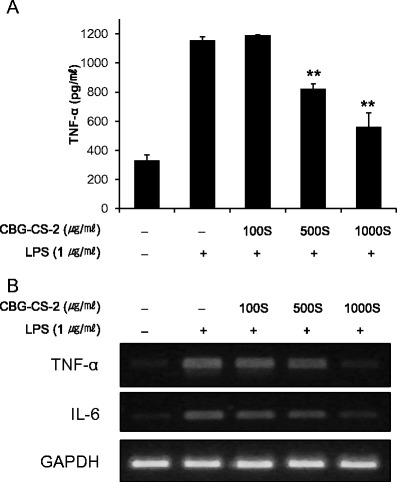
Effects of CBG-CS-2 on LPS-induced pro-inflammatory cytokine production in macrophage Raw264.7 cells. Cultures were treated with several concentrations (100S, 500S, and 1000S μg/ml) of CBG-CS-2, with LPS (1 μg/ml). **a** TNF-α secretion was measured in culture media using an ELISA kit. The results are expressed as mean with SEM from three independent experiments. Double asterisks (**) represent *P* < 0.01, Student’s *t*-test. **b** The mRNA expression of the pro-inflammatory cytokines, TNF-α and IL-6, was analyzed via RT-PCR. The GAPDH (glyceraldehyde 3-phosphate dehydrogenase) gene was used as a control

To analyze whether CBG-CS-2 has an effect on the pro-inflammatory cytokine, TNF-α, its secretion and expression in macrophages were measured using cytokine ELISA kits and RT-PCR. Treatment of LPS-activated cells with CBG-CS-2 led to significantly reduced secretion of TNF-α in Raw264.7 macrophages in a dose-dependent manner (Fig. 3).

### CBG-CS-2 reduced the activities of NF-κB and AP-1 in macrophages

**Fig. 4:**
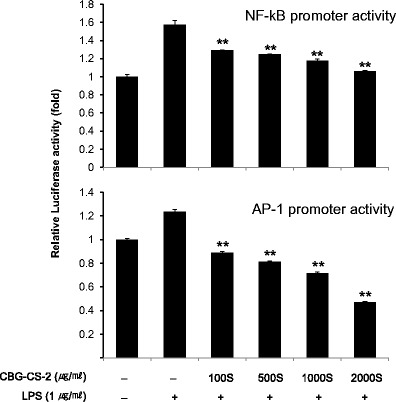
CBG-CS-2 inhibited LPS-induced NF-kB activation and AP-1 activity in macrophage Raw264.7 cells. Raw264.7 macrophages transfected with pGL3-NF-kB-Luc or pGL3-AP-1-Luc were stimulated with LPS (1 μg/ml) for 1 h with or without a variety of concentrations of CBG-CS-2 (100S, 500S, 1000S, and 2000S μg/ml). Lysates of these cells were subjected to a luciferase assay. The results are expressed as mean with SEM from three independent experiments. Double asterisks (**) represent *P* < 0.01, Student’s *t*-test

Since our results indicated that CBG-CS-2 affects iNOS expression and TNF-α secretion, we focused our interest on the two pivotal transcription factors critical in iNOS and TNF-α induction, i.e. NF-κB and AP-1 (Xie, Kashiwabara et al. 1994; Kristof, Marks-Konczalik et al. 2001). To investigate whether CBG-CS-2 could affect the activities of NF-κB and AP-1, a luciferase assay was conducted using macrophage cell lysates. CBG-CS-2 in fact significantly reduced the LPS-induced activities of both NF-κB and AP-1 (Fig. 4).

## Discussion

In this study we report that CBG-CS-2 reduces LPS-induced NO accumulation and inflammatory cytokine secretion in Raw264.7 cells. In this study, CBG-CS-2 seems to mediate NO reduction via inhibition of iNOS expression due to inhibited activation of NF-κB and AP-1.

The inhibition of iNOS in macrophages represents an important pathological mechanism in diverse inflammatory processes. Therefore, the regulatory mechanism of NO production represents a potential target for pharmacological intervention. In this context it is interesting that pretreatment of macrophages with CBG-CS-2 attenuates LPS-induced NO production.

*Cordyceps* (CS) is a traditional Chinese herb with various effects, including immune modulation (Li, Chiang et al. 2009). These mushrooms are known to modulate immune responses (Yang, Chen et al. 1999; Kuo, Tsai et al. 2001) and are expected to be effective in treating immune-related diseases. The therapeutic effects of these mushrooms, such as suppression of autoimmune diseases and allergy, have often been associated with their immunomodulatory effects (Shin, Lim et al. 2001; Shin, Lim et al. 2003). *Paecilomyces hepiali* (PH) is a derivative of *Cordyceps sinensis*, a fungus that has been shown to have anti-cancer and pro-apoptotic effects (Thakur, Hui et al. 2011).

Despite these reports on the inhibitory potential of *Cordyceps* toward immune modulation, none of the previous works were able to conclusively characterize the mechanisms responsible for PH-mediated anti-inflammatory effects in macrophages.

In order to evaluate the anti-inflammatory capacity of PH, we analyzed NO production, cytokine (TNF-α) secretion, and iNOS expression in LPS-induced macrophages with or without CBG-CS-2. Our present investigation clearly shows that CBG-CS-2 is an effective protector against NO generation. Importantly, our investigations provide evidence that CBG-CS-2 most likely attenuates NO production via its inhibitory action on iNOS induction. Nitric oxide synthase induction is predominantly regulated by the two pro-inflammatory transcription factors, NF-κB and AP-1 (Xie, Kashiwabara et al. 1994; Kristof, Marks-Konczalik et al. 2001). TNF-α is likewise predominantly regulated by NF-κB and AP-1 (Rhoades, Golub et al. 1992).

Due to the observation that CBG-CS-2 inhibits the expression of iNOS and TNF-α, we evaluated the level of two pro-inflammatory transcription factors, NF-κB and AP-1, Interestingly, CBG-CS-2 reduces the activation of NF-κB and AP-1. We here demonstrate for the first time that PH (CBG-CS-2) reduces the LPS-induced activation of NF-κB and AP-1 in Raw264.7 macrophages.

In summary, we demonstrated that CBG-CS-2 in pharmacologically relevant doses reduces the expression of crucial inflammatory mediators, i.e. iNOS and TNF-α, in Raw264.7 macrophages. This inhibitory action most likely occurs at the transcriptional level due to interference with the transcription factors, NF-κB and AP-1. This action of CBG-CS-2 may contribute to the anti-inflammatory potential of this biologically active mushroom.
